# Neural Induction Potential and MRI of ADSCs Labeled Cationic Superparamagnetic Iron Oxide Nanoparticle In Vitro

**DOI:** 10.1155/2018/6268437

**Published:** 2018-02-14

**Authors:** Weiqiong Ma, Qi Xie, Baolin Zhang, Huixian Chen, Jianyi Tang, Zhengxian Lei, Minyi Wu, Dingxuan Zhang, Jiani Hu

**Affiliations:** ^1^Medical Imaging Department, Nan Sha Center Hospital, Guangzhou Municipal First People's Hospital, Guangzhou Medical University, The Second Affiliated Hospital of South China University of Technology, Guangzhou, Guangdong 511457, China; ^2^Department of Radiology, Huizhou Municipal Central Hospital, Huizhou, Guangdong 516001, China; ^3^School of Materials Science and Engineering, Guilin University of Technology, Guilin, Guangxi 541004, China; ^4^Karmanos Cancer Institute, Wayne State University, 3990 John R. Street, Detroit, MI 48201, USA

## Abstract

Magnetic resonance imaging (MRI) combined with contrast agents is believed to be useful for stem cell tracking in vivo, and the aim of this research was to investigate the biosafety and neural induction of SD rat-originated adipose derived stem cells (ADSCs) using cationic superparamagnetic iron oxide (SPIO) nanoparticle which was synthesized by the improved polyol method, in order to allow visualization using in vitro MRI. The scan protocols were performed with T2-mapping sequence; meanwhile, the ultrastructure of labeled cells was observed by transmission electron microscopy (TEM) while the iron content was measured by inductively coupled plasma-atomic emission spectrometry (ICP-AES). After neural induction, nestin and NSE (neural markers) were obviously expressed. In vitro MRI showed that the cationic PEG/PEI-modified SPIO nanoparticles could achieve great relaxation performance and favourable longevity. And the ICP-AES quantified the lowest iron content that could be detected by MRI as 1.56~1.8 pg/cell. This study showed that the cationic SPIO could be directly used to label ADSCs, which could then inductively differentiate into nerve and be imaged by in vitro MRI, which would exhibit important guiding significance for the further in vivo MRI towards animal models with neurodegenerative disorders.

## 1. Introduction

Stem cell transplantation in treatment of central nervous system (CNS) diseases has the capacity to replace damaged nerve tissues so as to halt the progression of diseases and restore their physiological functions and then ultimately create favorable conditions for the clinical treatment [1–3]. However, stem cell biology remains incompletely understood despite significant advances in the field of neuroscience, including their migration, homing, survival, proliferation, and directed differentiation in vivo. Cell imaging has provided a feasible platform to solve the above-mentioned problems [4–6].

**Figure 1:**
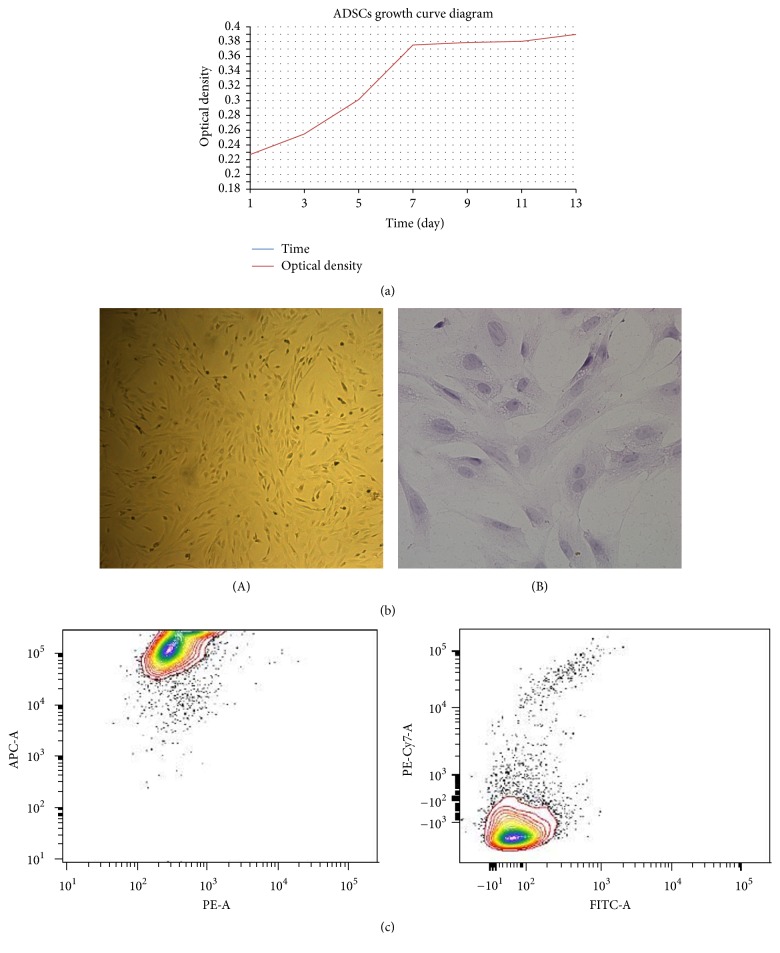
(a) The growth curve of ADSCs was appearing as a reverse “S”-shape. (b) The P_3_ ADSCs ((A) ×100; (B) ×HE-400) were appearing fusiform and spindle growth, and the projections were interconnected and fused. (c) The expressions of ADSCs' surface antigens: APC-CD29: strongly positively expressed, close to 100%; PE-CD44: positively expressed, approximately 30%–40%; PE/Cy-CD106 and FITC-CD45: negatively expressed, less than 5%.

**Figure 2:**
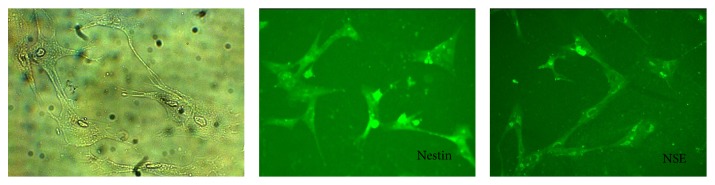
The microscopy revealed that the cell body turned round after induction, with several dendritic structures surrounded; the cells were similar to the pyramidal neurons, and the cellular immunofluorescence exhibited obvious expressions of NSE and nestin.

**Figure 3:**
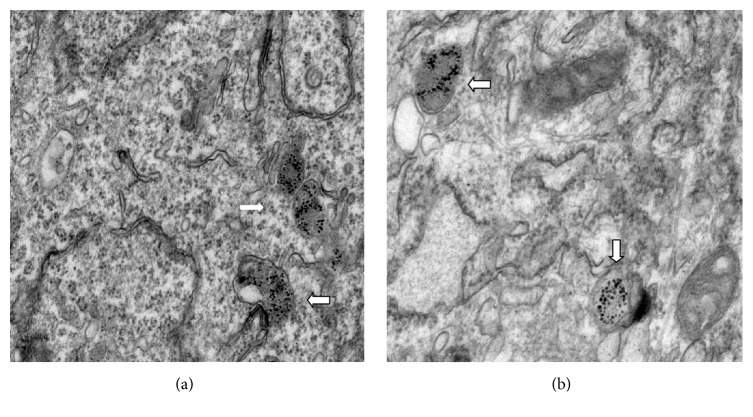
The absorbed SPIO nanoparticles mainly dispersed inside the perinuclear lysosomes (white arrows), and the phenomenon in the PEG/PEI-modified SPIO-labeling group (a) was more obvious than the PEG/PVP-modified SPIO-labeling group (b); the intracellular organelle structures were complete and not significantly damaged (×30000).

Cell imaging has used superparamagnetic nanoparticles coupled with magnetic resonance imaging to follow the fate of labeled cells in the organs and tissues. Therefore, it has become one of the most ideal analytical techniques of molecular imaging [7, 8]. The first widely applied strategy was to combine the commercial iron oxide nanoparticles (e.g., Feridex® or Resovist®) with the commercial transfection agents (e.g., liposomes, polylysine, or protamine sulfate) for the efficient labeling, but such shortcomings as imprecise surface properties and noncompletely consistent sizes of combinations influenced the results of tracing and imaging [9, 10]. Thus, various strategies had been explored to improve the stability of superparamagnetic nanoparticles. Laurent et al. [11] found that thermal decomposition in organic solvents could produce nanoparticles with precise control of charge and size relative to the combinations with transfection agents. So our research team used “one-pot” thermal decomposition method, namely, improved polyol method, which had made the SPIO to be modified with surface coating such as PEG, PEI, and PVP during the synthesis procedures (Figure  1S, supplementary material), and the nanoparticles exhibited several advantages. First, the nanoparticle could be synthesized with inherent cation and consistent particle size (Figure 2S, supplementary material). Second, the nanoparticles could be easily dispersed in aqueous media and other polar solvents owing to the coating by hydrophilic polyol ligands. Finally, the relatively higher reaction temperature of this procedure favored particles with a higher crystallinity and therefore a stronger magnetism (Figure 3S, supplementary material) [12, 13]. We conjectured that this new type of SPIO with excellent characterization (inherent cation, consistent size, strong magnetism, etc.) might be more effective in the field of labeling and tracing stem cells which were negatively charged.

In this study, we present cationic SPIO (PEG/PEI-modified SPIO) labeled with SD rat-originated ADSCs, followed by the experiments of labeling efficiency, biocompatibility, neural differentiation, and in vitro MRI imaging, aiming to explore its feasibility towards stem cell labeling and make early preparations for its further in vivo MRI imaging of neurodegenerative disease. PEG/PVP-modified zero charge-coated SPIO, synthesized with the same method [12, 13], was also used for the comparison, aiming to explore the roles of cation(s) in cell labeling. Meanwhile, our study had used ICP-AES to quantify the lowest iron content that could be detected by MRI; to our knowledge, this is the first series study to explore this area.

## 2. Materials and Methods

### 2.1. Extraction, Purification, and Identification of SD Rat-Originated ADSCs

The inguinal adipose tissues were harvested from 4-week-old SD rats (purchased from Guangdong Medical Experimental Animal Center) under sterile conditions; type I collagenase (Sigma) was then added for 45-min agitating digestion at 37°C; the mixture was then repetitively centrifuged, and after discarding the supernatant, the cellular pellet (primary cells, P0) was resuspended in DMEM/F12 (Hyclone company) with 10% fetal bovine serum (Gibco) for consecutive passage and purification. The P3 cells were then detected in the surface antigens (CD29, CD45, CD44, and CD106, Biolegend) using FACScan flow cytometer (BD FASCanto™, USA).

### 2.2. ADSCs Labeling with SPIO

The PEG/PEI-modified SPIO (coated with positive charge(s)) and the PEG/PVP-modified SPIO (coated with zero charge), synthesized by the improved polyol method, were used [12, 13], and the characteristics of these two types of functionalized nanoparticles are summarized in Table 1.

The stock solution of SPIO was firstly diluted with the cell culture medium to form the tracing agents with different concentrations (0 ug/ml, 6 ug/ml, 12 ug/ml, 25 ug/ml, 50 ug/ml, and 100 ug/ml), which were then used for direct coincubation for different periods (6 h, 12 h, 24 h, and 48 h); the pretest repetitively detected the effectiveness and safety of cell labeling using such assays as Prussian blue staining, cell viability (trypan blue staining), and cell proliferation (MTT).

**Figure 4:**
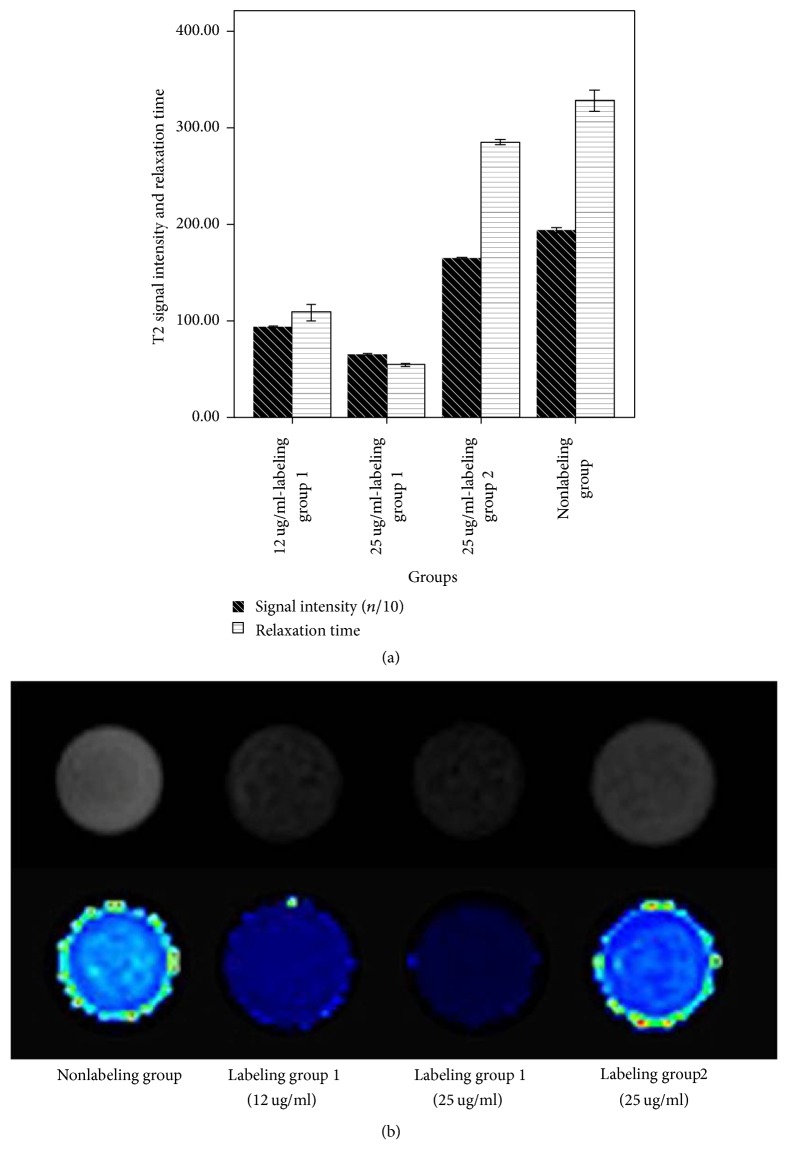
(a) The comparison of T2 signal intensity and relaxation time; (b) T2-mapping scanning among different groups.

Preexperiment had been carried out repetitively to detect the effectiveness and safety of cell labeling using such assays as Prussian blue staining (Figure  4S, supplementary material), cell viability (trypan blue staining), and cell proliferation (MTT) and then determined that the concentrations of PEG/PEI- and PEG/PVP-modified SPIO that could safely and effectively label ADSCs were 12 ug/ml, 25 ug/ml, and 25 ug/ml, and the incubation time was 12 h [14].

### 2.3. The Neural Induction Potential of Labeled Cells

10^6^ ADSCs were sampled, incubated with 25 ug/ml tracer for 12 h, and then set as the labeling group; 10^6^ unlabeled ADSCs were set as the control group. Preinduction culture medium (DMEM + 20% FBS + 1 mM *β*-mercaptoethanol) was added to these two groups for 24-h before incubation; after that, induction culture medium (DMEM + 5 mM *β*-mercaptoethanol) was replaced and cultured for another 5 h~5  days, during which period the cells' morphological changes were observed, and the neural markers (nestin and NSE, Santa Cruz Inc.) were detected.

### 2.4. TEM Observation

After fixation, dehydration, infiltration, and embedding, the cell precipitate was prepared for embedding slice, followed by conventional ultrathin slicing (70 nm) and 2-h lead-uranium staining. The cellular ultrastructures in the slices were then observed using TEM (JEM1230, Japan), including the locations of the SPIO nanoparticles and the integrities of mitochondria, endoplasmic reticulum, or Golgi apparatus.

### 2.5. In Vitro MRI

The 10^6^ ADSCs incubated with 12 ug/ml and 25 ug/ml PEG/PEI-modified SPIO and 25 ug/ml PEG/PVP-modified SPIO for 12 h were set as the labeling group, and another 10^6^ ADSCs were set as the nonlabeling group; the cells in these two groups were digested into single cell suspension with trypsin and then transferred into 0.8 ml EP tubes for centrifugation. The supernatant was discarded, and then 200 *μ*l of 5% gelatin was added to resuspend the cell precipitate; their T2WI signals and T2 values were then repetitively detected for 18 times.

The single cell suspension of ADSCs incubated with 25 ug/ml tracer for 12 h was dispersed into 0.8 ml EP tubes with 10^6^ cells/tube, 5 × 10^5^ cells/tube, 10^5^ cells/tube, 10^4^ cells/tube, and 10^3^ cells/tube, respectively; meanwhile, the nonlabeling group were also set to determine the minimum number of the labeled cells that MRI scanning could display.

10^6^ ADSCs incubated with 25 ug/ml tracer for 12 h were then washed with PBS, followed by continuous culture and passage. Each generation was then sampled with the same amount of cells for MRI scanning until no difference could be found with the nonlabeling group (10^6^ ADSCs); another same experimental group was simultaneously set up for the detection of iron content in single cell using ICP-AES.

Simens Vrio Tim 3.0T MRI scanner was used with animal-specific coil (5-cm in diameter). T2WI sequences were as follows: TR 2000 ms, TE 85 ms, FOV 220 mm × 220 mm, matrix 318 × 448, and layer thickness 1.5 mm; T2-mapping: TR 1000 ms, TE 13.8~69.0 ms, FOV 85.2 mm × 120 mm, matrix 318 × 448, and layer thickness of 1.5 mm.

### 2.6. Detection of Iron Content in the Labeled Cells Using ICP-AES

10^6^ ADSCs incubated with 25 ug/ml tracer for 12 h were placed in one 15 ml glass tube, and then 500 ul of 65–68% concentrated nitric acid (Guangzhou Donghong Chemical industry) was added to each sample. All the samples were placed into one oil-pan for 3-h high-temperature high-pressure reaction (121°C) until all the samples fully dissolved and exhibited uniformly no colour or light yellow (depending on the sample concentration). The standard samples for the quantitative detection by ICP-AES (Thermo Fisher, USA) included blank, 0.1, 1, 10, 50, and 100 (PPm), and according to the quantitative results, the standard curve could be obtained for the further calculation of unicellular iron content.

### 2.7. Statistical Analysis

SPSS17.0 statistical software was used for statistical analysis; the signal comparison between the labeling and the nonlabeling group, as well as between the relaxation times, used the two-independent-sample *t*-test; the labeling longevity comparison between the labeling and nonlabeling group used ANOVA, with *P* < 0.05 considered as significant difference.

## 3. Results

### 3.1. Extraction, Purification, and Identification of SD Rat-Originated ADSCs

The passage 0 (P_0_) cells began wall-adherent growth when cultured for 24–48 h, grew rapidly about 3–5 days later, and reached the peak on the 7th day. After that, the growth entered the plateau period, appearing as a reverse “S”-shaped growth curve (Figure 1(a)). The cells' morphologies became uniform when consecutively subcultured to passage 3 (P_3_), and interconnection of spindle projections and fusion growth appeared (Figure 1(b)). Flow cytometric analysis showed that the P3 ADSCs exhibited strongly positive expression of CD29 (reaching more than 95%), positive expression of CD44 (about 30%–40%), and negative expression of CD106 and CD45 (less than 5%) (Figure 1(c)).

### 3.2. The Neural Induction Potential of Labeled ADSCs

After neural induction, the labeled cells exhibited significant expressions of NSE and nestin; under a fluorescence microscope, cone and polygonal cells with green fluorescence could be seen, and projections with various lengths could be seen along the edge of the cell body, which tended towards the shape of pyramidal neurons (Figure 2).

### 3.3. TEM Observation

Electron microscope studies revealed that the absorbed nanoparticles dispersed inside lysosomes, vesicle-like aggregation appeared, and no nanoparticles were seen in nuclei. The chromatins were uniform, and the cell structures were basically intact, indicating that the absorbed nanoparticles did not significantly influence the morphology and structure of the cells (Figure 3).

### 3.4. In Vitro MRI

#### 3.4.1. T2-Mapping Scanning of the Labeling Group and the Nonlabeling Group

After labeled with the PEG/PEI-modified SPIO (labeling group 1) and the PEG/PVP-modified SPIO (labeling group 2), T2 signal intensity and T2 relaxation time of the inter- and intragroup showed significant differences (*P* < 0.01). T2WI signal intensity was significantly reduced and the T2 relaxation time was significantly shortened between the labeling group and the nonlabeling group, between labeling groups 1 and 2, and between the 25 ug/ml-labeling group 1 and the 12 ug/ml-labeling group 1 (Figures 4(a) and 4(b)).

#### 3.4.2. Labeling Rate of SPIO

**Figure 5:**
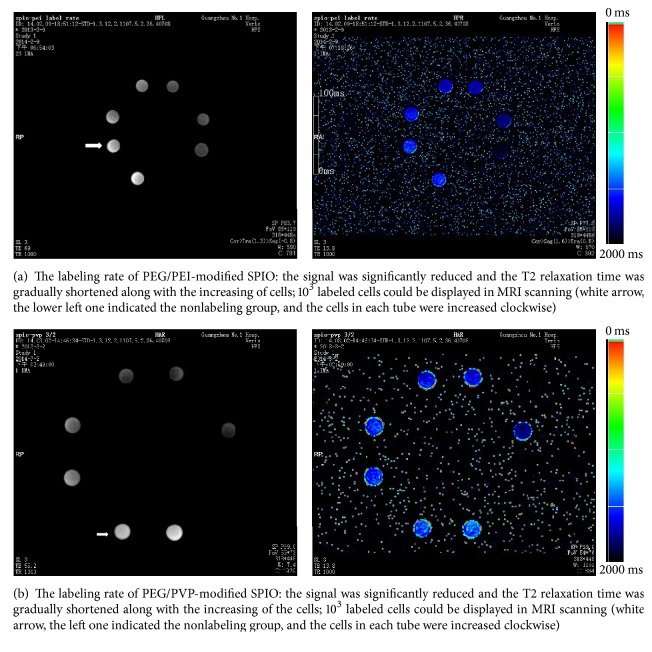


It could be seen from the T2-mapping images that the signal was significantly reduced and the T2 relaxation time was gradually shortened along with the increasing of the cells. 10^3^ labeled cells could be displayed in MRI scanning (Figures 5(a) and 5(b)).

#### 3.4.3. Labeling Longevity of SPIO

**Figure 6:**
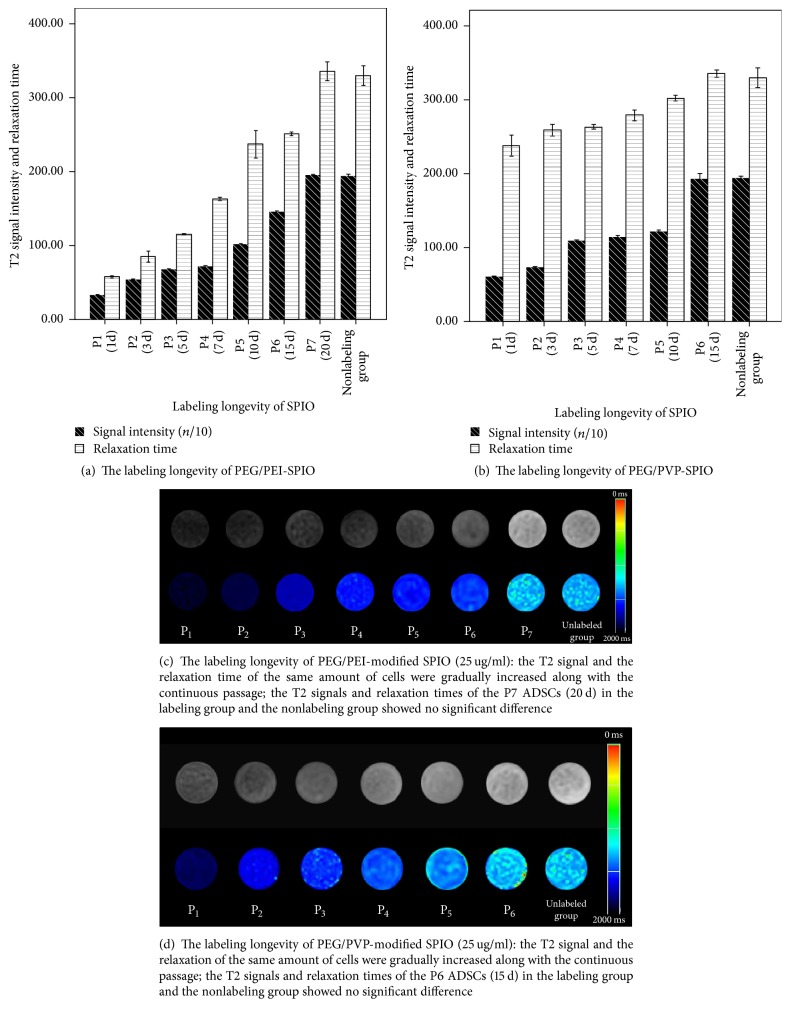


On the T2-mapping images, the T2 signals and the relaxation time of the same amount of cells labeled with PEG/PEI-modified SPIO in different generation (25 ug/ml, passaged for 1 day, 3 days, 5 days, 7 days, 10 days, 15 days, and 20 days) were gradually increased with the extension of cell growth and proliferation time, and the T2 signal and relaxation time of passage 7 (P7) ADSCs (20 d) showed no significant difference between the labeling group and the nonlabeling group (*F* = 0.113, *P* = 0.740; *F* = 4.369, *P* = 0.051). Meanwhile, the T2 signals and the relaxation time showed no significant difference between the labeling group with the PEG/PVP-modified SPIO and the nonlabeling group in passage 6 (P6) ADSCs (15 d) (*F* = 1.891, *P* = 0.186; *F* = 3.682, *P* = 0.071, Figures 6(a)–6(d)).

#### 3.4.4. Unicellular Iron Content in the Labeling Cells

With the extension of cell growth and proliferation, the unicellular iron content in the labeling cells was decreased. When the T2WI signal and the T2 value showed no significant difference, the intracellular iron content was 1.56~1.8 pg/cell by ICP-AES method (Table 2).

## 4. Discussion

It was reported that the commercial SPIO carried negative charge(s) on its surface, so commercial transfection agent which carried positive charge(s) must link to stem cells for effective labeling, but this strategy would result in inaccurate surface properties as well as inconsistent particle sizes, thus causing the discrepancy in labeling effects [15, 16]. Therefore, this study used coating-modified SPIO of positive or zero charge to label ADSCs directly, and the results showed that these two types of SPIO could safely and effectively label ADSCs without transfection agent, maintaining relatively stable status in labeling. Furthermore, the positive-charged SPIO could more effectively label ADSCs than the zero-charged groups, indicating that cation(s) on the surface of SPIO nanoparticle played an essential role in ADSCs' labeling.

In this study, 12 h incubation of ADSCs with the cationic PEG/PEI-modified SPIO (within the concentration range 12–25 ug/ml) could achieve more than 95% labeling rate of iron particles, and the intracellular iron content might reach as high as 35.4 pg/cell. Compared to this new type of SPIO, previous commercial SPIO required the concentration range as 25–50 ug/ml and the incubation time as 18–24 h to achieve more than 95% labeling rate in mesenchymal stem cells, and the intracellular iron content could only be up to 15 pg/cell or 17.9 pg/cell [17, 18]. These results suggested that this cationic SPIO could much more quickly and effectively label the stem cells than previous commercial SPIO, mainly due to the PEI coating. PEI is a typical water-soluble cationic polymer with high positive charge which can interact with the negatively charged cell membranes and facilitates better cell internalization through endocytosis. However, the high positive charges can disrupt the cell membrane, resulting in toxicity to the cells [19, 20]. So our research team grafted PEG to PEI, which provided colloidal stability and neutralized the toxic effects of the PEI [12, 14].

Safety is the primary premise of SPIO-labeling towards stem cells. In this study, the cytotoxicity of ADSCs caused by this cationic SPIO in the incubation concentration and time which could achieve labeling efficiency was investigated. The result showed that the concentrations of PEG/PEI-modified SPIO that could safely and effectively label ADSCs were 12–25 ug/ml, and the incubation time was 12 h, showing no significant change in cell viability and proliferation between labeling and nonlabeling group. TEM revealed that the labeled cells exhibited no obvious organelle damage. All these results indicated that this effective incubation concentration and time did not significantly inhibit the biological activities of stem cells. The labeling safety should not only assess whether the tracer agent would inhibit the biological activities of the host cells but also consider whether the deposition area of the absorbed nano-iron particles could precipitate an effective metabolic degradation. Previous studies generally considered that the nanoparticles were transferred into the stem cells via endocytosis, then the nanoparticles localized in the cytoplasm instead of entering the nuclei by forming the early stage of endosome-like vesicles [21, 22]. Some studies found that the nanoparticles positioned in the late endosomes or secondary lysosomes of the cytoplasm [23, 24]. Therefore, it was presumed that while the nanoparticles were internalized into the cytoplasm of the lysosomal compartment from early endosomes, then they might gradually be delivered to late endosomes and lysosomes around the nuclei with the release of the vesicles and the growth of the endosomes [25, 26]. In this study, the ultrastructural observation of labeled cells by TEM indicated that the internalized SPIO was located inside early endosomes and lysosomes at the region of cytoplasm, which was consistent with previous studies. Due to the existence of a large number of molecular proteins (ferritin, transferrin, and hemosiderin, etc.) in living tissues, the internalized iron in lysosomes might be stored or degraded by further hemoglobin synthesis, thus maintaining the iron metabolism balance in vivo [27, 28]. It might be more beneficial in avoiding potential cytotoxicity as the absorbed SPIO could be metabolized and degraded in lysosomes.

All procedures, applied for this study, were to explore the feasibility of stem cell therapy towards CNS disorders. Therefore, it should make clear that whether this labeling strategy would impact the neural induction of ADSCs. Referring to the study of Zuk et al. [29] and Lin et al. [30], *β*-mercaptoethanol was coincubated with ADSCs in the labeling and nonlabeling group. The microscopic observation showed that both of them exhibited the similar morphologies of pyramidal neurons, and the expressions of neural markers (nestin and NSE) were positive, among which NSE was more obviously expressed. The expression of nestin proved that ADSCs entered the key step of neural differentiation. Enolases include three homotypes (namely, *α*, *β*, and *γ*), and the *γ*-type enolase, namely, NSE, is expressed in the nervous tissues and secreted by the neurons. Therefore, the NSE expression proved that ADSCs differentiated and proliferated towards the neurons, and the feasibility of ADSCs differentiating towards the neurons was thus demonstrated. The neural induction potentials between the labeling and nonlabeling group showed no significant difference, indicating that SPIO-labeling would not significantly impact the neural induction potential of ADSCs, reflecting the safety of the SPIO-labeling indirectly.

Besides the labeling safety and effectiveness, effective relaxation and as-long-as-possible labeling durability should be required to achieve better dynamic visualization imaging of the transplanted stem cells. The SPIO-labeled ADSCs in this study showed sensitive and effective relaxation; 10^3^ cells could cause the changes of the T2 signal and relaxation time. The T2 signal negative effects were also enhanced with the increasing of the incubation concentrations, which was consistent with previous studies using certain commercial SPIO [15, 31]. The other finding of this research was that the signal intensity and the relaxation time in the cationic PEG/PEI-modified SPIO group (12 ug/ml) were significantly lower and shorter than those in the PEG/PVP-modified SPIO group (25 ug/ml). Even more, the iron content detected with the ICP-AES could reach 20.16 pg/cell in the former group, but only 6.96 pg/cell in the latter group. Surmising that the stem cell surface is negatively charged, the surface charge would play an important role in the intracellular transportation of exogenous nanoparticles [32]. Therefore, cationic PEG/PEI-modified SPIO nanoparticles could be absorbed and lead to internalization more easily than zero charge-coated PEG/PVP-modified SPIO ones and then achieve greater relaxation performance.

Liu et al. [17] found the labeling longevity of Resovist-PLL complexes towards marrow mesenchymal stem cells (MSCs) could generally last up to 20 d, and even certain study found the detection of human marrow mesenchymal stem cells (hMSCs) with MRI was possible up to 35 d [33]. In this study, the labeling longevity of PEG/PEI-modified SPIO towards ADSCs could last up to 20 d, while the PEG/PVP-modified SPIO only last up to 15 d, showing no distinct advantage. Supposing that the proliferation of the cells inhibited the SPION from retaining inside the cells, some studies had shown that the proliferation of ADSCs was significantly greater than bone marrow mesenchymal stem cells (BMSCs) [34, 35], which might be the reason that the labeling longevity of PEG/PEI-modified SPIO towards ADSCs in this study was shorter than that of commercial SPIO towards hMSCs. In addition, the exocytosis of the cells which was enhanced as the decreasing of the particle size could also dilute the intracellular iron content. Xu et al. [36] added PLGA particles into SPIONs to change the diameter of the nanoparticles and found that these PLGA-SPIONs could significantly increase the labeling longevity towards MSCs because the particle diameters of Feridex and Resovist were bigger than SPIO synthesized by an improved polyol method, which would extend detection time with MRI at some degree. The internalized iron would be evenly or unevenly dispersed into two daughter cells along with the cell proliferation, as passed through several cell cycles, and the iron content would be gradually diluted to the level lower than the MRI detectability. Previous studies did not specifically point out the detection limit of intracellular iron content by MRI. Our study had found that the T2 value showed no difference with the nonlabeling group when the iron content in labeling ADSCs cell was reduced to 1.56~1.8 pg/cell, quantifying the lowest iron content that could be detected by MRI.

ADSCs, which had wide source ranges and easy sampling procedures, were selected for the labeling in this study; the data from only one species were relatively limited. In further studies, different species of stem cells should be selected for the detection so as to better serve the field of stem cell labeling. Secondly, this study performed neural induction and MRI imaging in vitro towards the labeled stem cells, which belonged to one relatively early basic research. In order to achieve the integrity of the study, the next step should be focused on the biodistribution of the nanoparticles by using the accumulative dynamics of the nanoparticles in various animal model systems in vivo MRI.

## 5. Conclusions

The results of this study showed that the modified SPIO synthesized by an improved polyol method could be directly used to label ADSCs, safely and effectively. Labeling ADSCs could be inducted to differentiate into nerve and be imaged with MR in vitro. The surface charge(s) of nano-iron particles played an extremely important role in cell labeling. Meanwhile, the study also quantified the lowest iron content in labeling cell with MRI tracing imaging. Therefore, this cationic SPIO could be used for stem cell tractography, which would exhibit important guiding significance for the further in vivo MRI towards animal models with neurodegenerative disorders.

## Figures and Tables

**Table 1:** The characteristics of the two types of functionalized nanoparticles.

Agent	Coating	Hydrodynamic size (nm)	Zeta potential (mV)
PEG/PVP-SPIONs	PEG, PVP	18–22	0
PEG/PEI-SPIONs	PEG, PEI	19–24	25 ± 1.5

**Table 2:** The detection of iron content in labeling cells by ICP-AES (pg/cell).

	P1	P2	P3	P4	P5	P6	P7
*Labeling group 1*							
12 *μ*g/ml	20.16	9.36	7.32	3.96	2.04	1.88	1.56
25 ug/ml	35.4	17.04	14.4	6.72	3.12	2.60	1.80
*Labeling group 2*							
25 ug/ml	5.52	3.36	3.12	2.64	2.64	1.56	

## Abbreviation

ADSCs: Adipose derived stem cells
CNS: Central nervous system
MRI: Magnetic resonance imaging
SPION: Superparamagnetic iron oxide nanoparticles
PEG: Polyethylene glycol
PVP: Polyvinylpyrrolidone
ICP-AES: Inductively coupled plasma-atomic emission spectrometry
NSE: Neuron specific enolase
BMSCs: Bone marrow mesenchymal stem cells
hMSCs: Human marrow mesenchymal stem cells
SPIO: Superparamagnetic iron oxide
PEI: Polyethyleneimine
TEM: Transmission electron microscopy.

## Supplementary Materials

Supplementary Materials Figure  1S: schematic representation of the synthesis and probable coating of the magnetite nanoparticles. Figure  2S: the magnetite nanoparticles coated with PEG/PVP or PEG/PEI dissolved in water: (a) hydrodynamic size analysis and (b) zeta potential measurements, showing inherent cation and consistent particle size. Figure  3S: M-H curve of the magnetite nanoparticles by the thermal decomposition of Fe(acac)3 in PEG containing PEI and PVP, showing stronger magnetism. Figure  4S: the Prussian blue images of ADSCs incubated with 25 *μ*g/ml PEG/PEI-SPIONs (a) and 25 *μ*g/ml PEG/PVP-SPIONs (b). Prussian blue staining showed more iron content in PEG/PEI-SPIONs labeled groups than that in PEG/PVP-SPIONs labeled groups.
