# Supplementary material for: Neural Induction Potential and MRI of ADSCs Labeled Cationic Superparamagnetic Iron Oxide Nanoparticle In Vitro
Source: Contrast Media Mol Imaging. 2018 Feb 14;2018:6268437. doi: 10.1155/2018/6268437 (PMC5832102; doi:10.1155/2018/6268437)
Supplement: Supplementary Materials — Figure 1S: schematic representation of the synthesis and probable coating of the magnetite nanoparticles. Figure 2S: the magnetite nanoparticles coated with PEG/PVP or PEG/PEI dissolved in water: (a) hydrodynamic size analysis and (b) zeta potential measurements, showing inherent cation and consistent particle size. Figure 3S: M-H curve of the magnetite nanoparticles by the thermal decomposition of Fe(acac)3 in PEG containing PEI and PVP, showing stronger magnetism. Figure 4S: the Prussian blue images of ADSCs incubated with 25 μg/ml PEG/PEI-SPIONs (a) and 25 μg/ml PEG/PVP-SPIONs (b). Prussian blue staining showed more iron content in PEG/PEI-SPIONs labeled groups than that in PEG/PVP-SPIONs labeled groups. [file 6268437.f1.docx]

**Supplementary Materials**

**
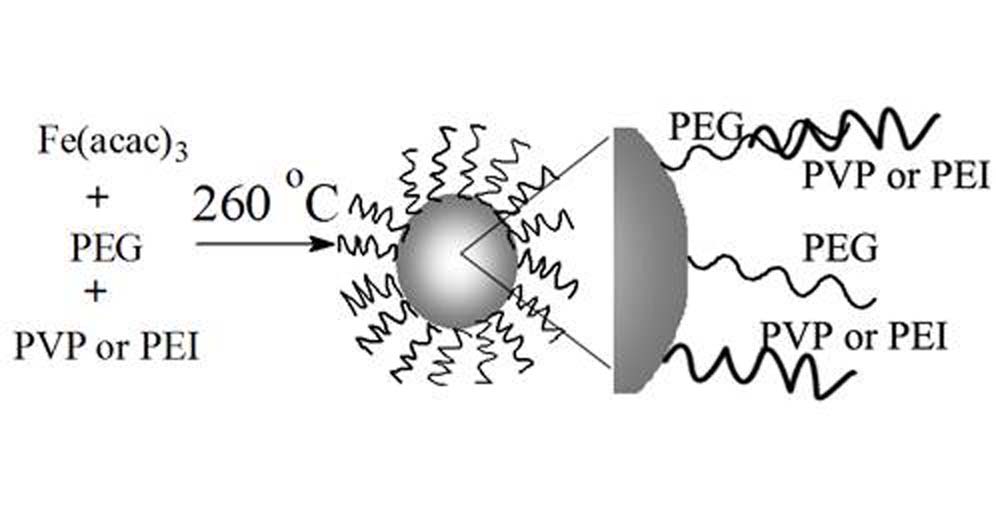
**

Fig 1. Schematic representation of the synthesis and probable coating of the magnetite nanoparticles.


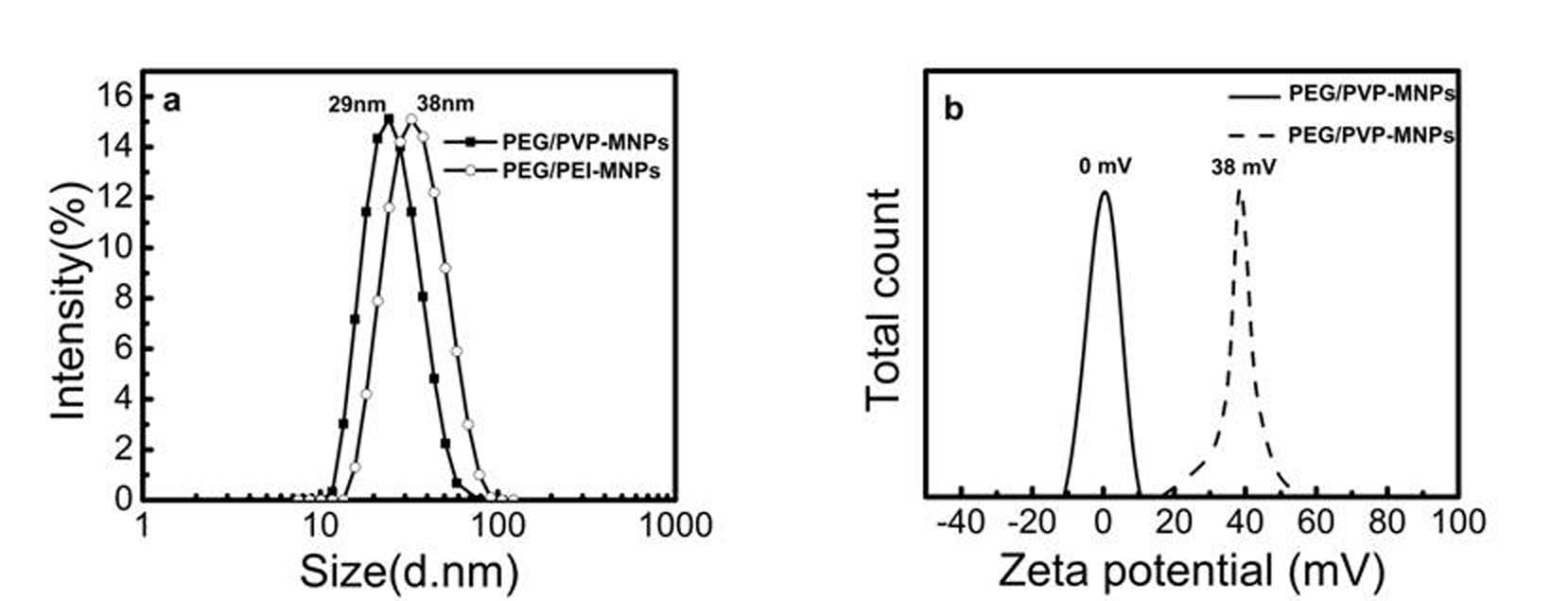


Fig 2. The magnetite nanoparticles coated with PEG/PVP or PEG/PEI dissolved in water: (a) hydrodynamic size analysis and (b) zeta potential measurements,showing inherent cation and consistent particle size.

**
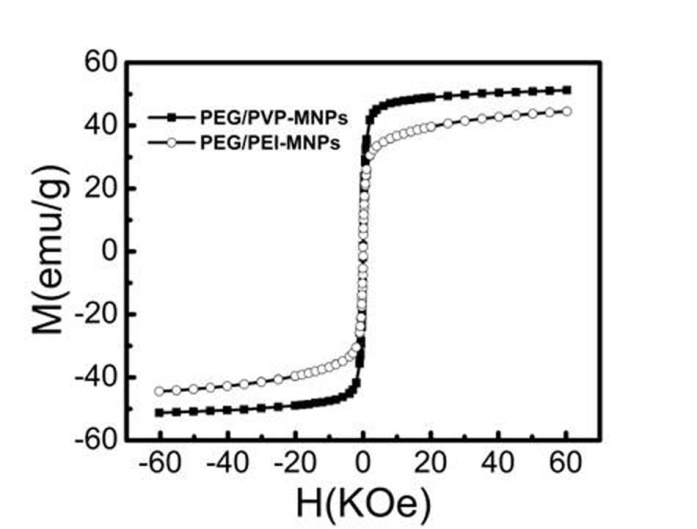
**

Fig 3. M–H curve of the magnetite nanoparticles by the thermal decomposition of Fe(acac)_3_ in PEG containing PEI, PVP,showing stronger magnetism.

**
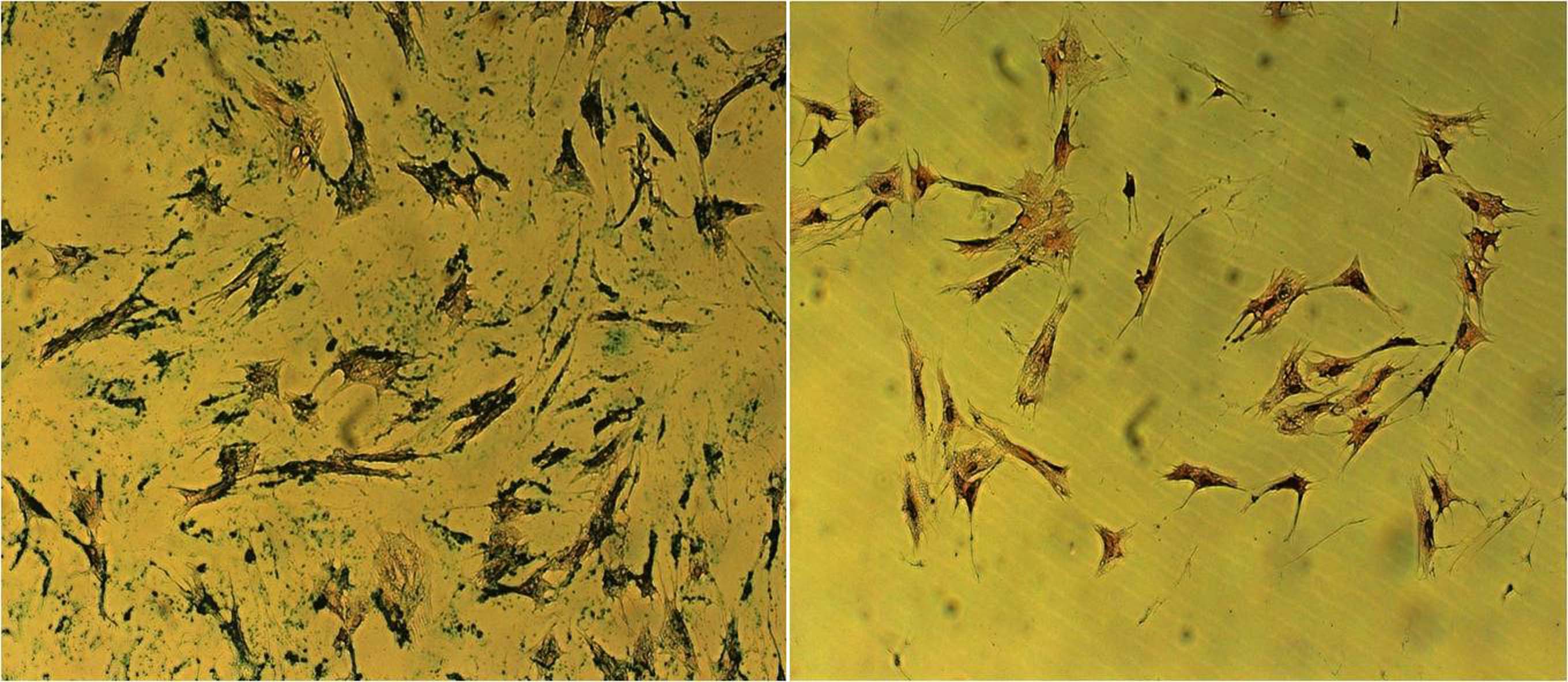
**

**(a) (b)**

Fig 4. The Prussian blue images of ADSCs incubated with 25 μg/ml PEG/PEI-SPIONs (a) and 25 μg/ml PEG/PVP-SPIONs (b). Prussian blue staining showed more iron content in PEG/PEI- SPIONs labeled groups than that in PEG/PVP-SPIONs labeled groups.
